# Followup in Southern California: Decreased Birth Weight following Prenatal Wildfire Smoke Exposure

**DOI:** 10.1289/ehp.120-a362b

**Published:** 2012-08-31

**Authors:** Rebecca Kessler

**Affiliations:** Rebecca Kessler, based in Providence, RI, writes about science and the environment for various publications. She is a member of the National Association of Science Writers and the Society of Environmental Journalists.

In fall 2003 wildfires blazed in Southern California, sending pollutant-laden smoke directly into the heavily populated Los Angeles and Orange counties. A new study of birth records from the area shows a decreased average birth weight among infants carried *in utero* during the wildfires **[*EHP* 120(9):1340–1345; Holstius et al.]**.

Chronic maternal exposure to ambient particulate matter (PM) and indoor biomass smoke during pregnancy has been linked to decreased infant birth weight, although similar effects attributable to wildfire smoke have been little studied. In this study, researchers analyzed 886,034 birth records of babies delivered between 2001 and 2005 in the South Coast Air Basin, an area that overlaps the wildfire-affected regions. They compared the average birth weight of infants carried *in utero* during the wildfires with that of infants who were either born before or conceived after the wildfires. They also compared birth weights of infants exposed during the first, second, and third trimesters of pregnancy.

After adjusting for infant sex, gestational age at birth, and other factors known to influence birth weight, the researchers found that exposed infants weighed an average of 6.1 g (0.2 oz) less at birth than unexposed infants. Infants exposed during the second trimester showed the largest average reduction, at 9.7 g. Infants exposed during the third trimester showed an average reduction of 7.0 g, and those exposed during the first trimester showed an average reduction of 3.3 g (the latter was not statistically significant). The trends were corroborated by a sensitivity analysis that compared pregnant mothers according to whether the air-pollution monitor nearest their residence recorded high or low PM levels during the wildfires.

**Figure:**
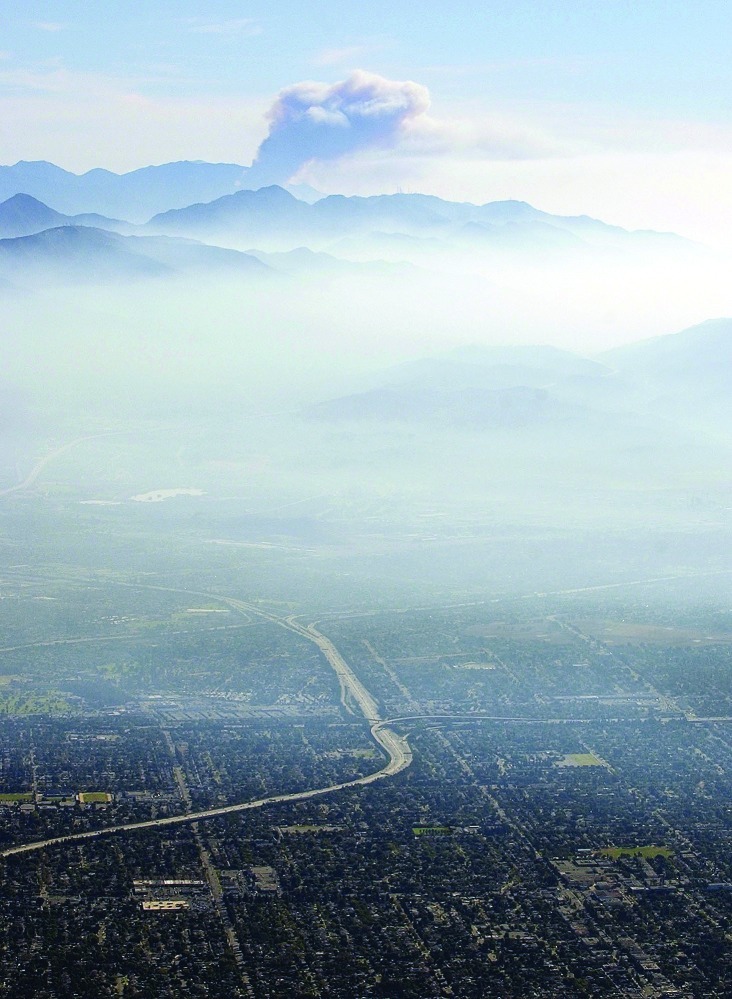
Smoke from wildfires burning across Southern California, 27 October 2003 AP Photo/Kevork Djansezian

The small reductions in birth weight attributed to the wildfires are unlikely to have serious health effects in any given individual. However, wildfires are predicted to increase in frequency and severity as climate change progresses, and the researchers note that the proportion of low-birth-weight babies—those weighing less than 2,500 g (5.5 lb) at birth—could rise as a result. This could have important populationwide repercussions because these babies are at increased risk for a variety of poor health outcomes.

PM and carbon monoxide are among pollutants released by wildfires that could plausibly contribute to the birth-weight decrease. However, the researchers note that other pathways are possible and worthy of investigation, among them the increased stress that living through a wildfire may place on pregnant women.

